# Mitochondrial Replacement Therapy: In Whose Interests?

**DOI:** 10.1017/jme.2022.98

**Published:** 2022

**Authors:** Forough Noohi, Vardit Ravitsky, Bartha Maria Knoppers, Yann Joly

**Affiliations:** 1. CENTRE OF GENOMICS AND POLICY, DEPARTMENT OF HUMAN GENETICS, McGILL UNIVERSITY, MONTREAL, QUEBEC, CANADA; 2. DEPARTMENT OF MEDICINE, UNIVERSITY OF CHICAGO, CHICAGO, UNITED STATES (CURRENT AFFILIATION); 3. SCHOOL OF PUBLIC HEALTH, UNIVERSITY OF MONTREAL, MONTREAL, CANADA

**Keywords:** Mitochondrial Replacement Therapy, MRT, Reproductive Autonomy, Genetic Relatedness

## Abstract

Mitochondrial replacement therapy (MRT), also called nuclear genome transfer and mitochondrial donation, is a new technique that can be used to prevent the transmission of mitochondrial DNA diseases. Apart from the United Kingdom, the first country to approve MRT in 2015, Australia became the second country with a clear regulatory path for the clinical applications of this technique in 2021. The rapidly evolving clinical landscape of MRT makes the elaboration and evaluation of the responsible use of this technology a pressing matter. As jurisdictions with less strict or non-existent reproductive laws are continuing to use MRT in the clinical context, the need to address the underlying ethical issues surrounding MRT’s clinical translation is fundamental.

In identifying the challenges of MRT’s clinical implementation, a key question concerns the legitimacy of using this technology to fulfill the parental interest in having and raising genetically related children free of mitochondrial DNA disease. Debates on the ethics of introducing new reproductive technologies are centered on the requirement of balancing possible benefits and risks and protecting the reproductive freedom of prospective parents. In this article, we focus on and respond to two objections to MRT: (1) MRT has limited social value as the technique is neither a “treatment” nor a “cure,” and (2) prospective parents have other reproductive alternatives such as egg and embryo donation, as well as adoption. We begin by presenting a brief review of the science of MRT. We then examine and respond to the objections to the parental “interest” in having genetically related children and the “therapeutic” nature of MRT. Finally, we highlight the tension between the applications of MRT: to prevent disease vs. to treat infertility, an area of societal interest regarding MRT requiring further exploration.

## Mitochondria: “Power Plants” of the Cell

Mitochondria are membrane-bound microscopic organelles responsible for cellular respiration (the breakdown of carbohydrate substrates in the presence of oxygen) and energy production. Mitochondria have their own DNA (mtDNA), separate from the nuclear DNA (nDNA). Mitochondrial DNA is typically a small circular double-stranded molecule containing 37 genes which encode the core components of the mitochondrial respiratory complexes.^1^ A given cell can house anywhere from one to hundreds of thousands of mitochondria, depending on the cell type, structure, and function.^2^

Mitochondrial DNA mutations are associated with a broad phenotypic spectrum, ranging from mild myopathies to devastating multisystem syndromes.^3^ It is estimated that 1 in 4300 people are affected by primary mitochondrial diseases.^4^ Mitochondrial disorders can be present at birth or manifest later in life. They cause debilitating physical, developmental, and cognitive impairments that are progressive. There is no cure for inherited mtDNA diseases, and for the vast majority of patients, therapy is limited to the early detection and alleviation of symptoms.^5^

## Mitochondrial Replacement Therapy (MRT)

Methods used for potentially diagnosing and preventing the transmission of mitochondrial disorders include prenatal diagnosis (PND) and preimplantation genetic diagnosis (PGD). These techniques, though, cannot benefit a wide range of women who are at risk of transferring harmful mitochondrial mutations to their descendants. PND could be invasive and if it was confirmed that the fetus had a mtDNA deficiency, parents would need to choose between continuing with the pregnancy or terminating it. PGD carries a certain degree of uncertainty in predicting the degree of mtDNA heteroplasmy transmitted to future children and the risk of disease manifestation.^6^

For families who wish to have children free of mitochondrial disease, egg and embryo donation, as well as adoption, are considered alternatives.^7^ For those who wish to have genetically related children free of mitochondrial disease, MRT could be a possible option. The most notable methods of MRT include Pronuclear Transfer (PNT) and Maternal Spindle Transfer (MST).^8^ PNT entails fertilizing a healthy donated egg and the prospective mother’s oocyte with the father’s sperms. Fertilized oocytes then develop until the early zygote stage. The pronuclei of the zygote formed by the donated oocyte is discarded and replaced by the intending mother and father’s pronuclei. In MST, the parental nDNA transfer happens before fertilization. This method requires discarding the metaphase II spindle from the donor oocyte. The mother’s spindle complex will then be delivered to the enucleated donor oocyte, followed by fertilization.^9^ It is important to recognize that clinics in countries where reproductive laws are less stringent or absent are already using MRT (e.g., Greece). To our knowledge, seventeen children have already been born using this technology.^10^

## Policy Developments

Thus far, the United Kingdom (UK) and Australia are the only countries that have adopted legislation with regard to the clinical implementation of MRT. In 2015, following an ethical assessment conducted by the Nuffield Council on Bioethics (2012), a public dialogue and deliberation carried out by the Human Fertilisation and Embryology Authority (HFEA) (2012-2013), a public consultation on draft regulations performed by the Department of Health (2014), and three separate reports on the safety and efficacy of MRT (2011, 2013, 2014) (HFEA 2021), the UK passed the *Human Fertilisation and Embryology (Mitochondrial Donation) Regulations*. The law mandates a case-by-case assessment of applications proposing to use MRT. The technique may only be used to prevent *serious* mitochondrial diseases.^11^ As of 4 January 2022, 27 applications have been approved for individual patient use of MRT by the HFEA Statutory Approvals Committee (SAC).^12^

In Australia, following the release of two reports (an expert statement on the science of MRT and a public consultation) by the National Health and Medical Research Council, the *Mitochondrial Donation Law Reform (Bill 2021)* was introduced into the Parliament in March 2020. On May 11, 2021, the Australian Government pledged $10.3M over 10 years to the implementation of MRT in research and clinical contexts in Australia. On March 30, 2022, the Bill was voted upon favorably in the Senate. At this time, the government will proceed with the first stage of implementing MRT in Australia and authorize the technique for specific training and research means, including clinical trials.^13^

## MRT and the Interest in Having Genetically Related Children

In the context of MRT, several objections have been raised in the bioethics literature, such as the slippery slope toward genetic modification, potential non-therapeutic uses, potential harms to egg providers, to future generations, or to society.^14^ The crux of the ethical argument in favor of MRT is the need to fulfill prospective parents’ strong interest in having and raising genetically related children free of mitochondrial disease. Over the last four decades, the interest in having genetically related children has been perceived as strong enough to justify a wide array of assisted reproduction technologies, such as IVF. It has been argued that this interest is deep and broad and that MRT extends this recognition and the reproductive autonomy of prospective parents.^15^ According to certain critics, considering the *need* to access MRT as a “compelling” one, overvalues genetic relatedness and devalues non-genetic familial ties: “if there is a compelling argument to be made it is an argument against human nuclear genome transfer on the grounds that a desire for genetically related children, though often interpreted as a need, is at most a want.”^16^ MRT, it is thus argued, ought not be a priority for society since alternatives, such as egg and embryo donation or adoption, do exist.^17^

The interest in having genetically related children may be considered a preference, a powerful desire, an interest, or even a right. Regardless of one’s view on this matter, to many, genetic relatedness matters and bears a strong, profound meaning. In the absence of a responsible MRT framework, women have to choose from a set of alternatives (i.e., egg and embryo donation, adoption, childlessness, or risk having an affected child). In the first qualitative interview study in Canada regarding MRT, Noohi et al. (2021) addressed the objective of MRT: having children free of mitochondrial diseases while maintaining genetic relatedness between mother and child. Of eight individuals affected by primary mitochondrial diseases who either had affected children or no children at all, seven maintained that they did not consider the said alternatives to MRT as viable options.^18^

I’m gonna be honest with you. I have a daughter. I don’t want her to live through what I lived through. God forbid if she doesn’t make it to her child-bearing years. If she does, she will be informed that if she wants a child of her own, there are countries that could help her. We have money aside for that. I know that it’s a very very sad thing to talk about, but I want her to have the opportunity to have and to carry a child of her own that will not land her in the hospital like I did. I think everybody is equally allowed to have that. […] and what’s great in her case is that she was born in 2011, she has a lot of time in front of her for this to become legal, or for research to advance. I didn’t, but that’s okay, we will deal with what it is now. But for her, there is a promising future, and a way to prevent it from continuing, right?

[…] I think it’s more psychological, and that you should have the right to have a family however way you want to. Whether you adopt, you have a surrogacy, you have a biological child… People who adopt are seen differently in society than people who have biological children. Not because it’s right or wrong. You will meet people who will never be able to love a child if they adopt [as if they were their own biological child]. People will present themselves and say yes, I would not be able to… I had this discussion with my husband. My husband would be like: I have two biological children now and if you tell me that I am going to adopt now, I can tell you for sure that I will never be able to love that child like my own kids.

[…] I would do anything to have a biological kid. I have always wanted my own kids, but I don’t have that kind of lifestyle that I can just quit my job and go to another country to get the process of MRT and get pregnant. If MRT doesn’t become legal [in Canada] by the time I am ready to have children, I am going to adopt or try other means of non-biological children by myself.

Mitochondrial disease patients’ profound interest in having genetically related children free of mitochondrial disease is rooted in deep generational pain and grief. Many mothers, having gone through the excruciating pain of having passed on debilitating mutations to their progeny, see MRT as a promising future that could grant their children’s children an equal opportunity at a chance of life without dreadful diseases.^19^

Indeed, in considering the clinical introduction of MRT, it is essential to consider the interests and the rights of all parties: children born of MRT, prospective parents, and egg donors. As recognized by the International Commission on the Clinical Use of Human Germline Genome Editing (2020), we believe the UK’s MRT policy is an example of a stepwise clinical introduction that future preventive interventions need to follow: (1) support from patient groups advocating in favor of the technology; (2) public engagement regarding ethical aspects; (3) safety and efficacy reviews conducted by independent expert panels; and (4) regulatory approval on a case-by-case basis.^20^

Choices related to human reproduction are central to personal autonomy and deeply rooted within our most profound individual nature. Article 16 of the *Universal Declaration of Human Rights* proclaims that “men and women of full age” “have the right to found a family.”^21^ An approach to reproductive autonomy that is broad in scope and attentive to context is crucial for preparing for a future in which ever-evolving technologies, such as MRT and CRISPR, continue to expand reproductive options.

Considering millions of children worldwide who could benefit from adoption, an argument against MRT states that parents seeking to use this technique should adopt, rather than create *new* life:

The reasons for wanting a genetic child do not defeat a *pro tanto* duty to adopt children instead, with a possible exception. These reasons are too trivial, presuppose the value of the genetic connection, are inappropriate in a normative parental context, or fail to make a relevant distinction between genetic and adopted children. A promising candidate for a one-time exception may be grounded in a woman’s strong desire to experience pregnancy.^22^

Adoption is indeed an excellent family-building choice for many people. However, as forty years of IVF have shown, it is not an option that provides a suitable alternative for all. The journey to adoption can be long and complex, entail financial and emotional burdens, and might not be completed successfully. A putative ‘moral duty to adopt’ ought to be considered a responsibility shared by *all persons* capable and willing to be parents, not solely those at-risk of transmitting serious genetic diseases to their progeny. Hence, prospective parents who suffer from mitochondrial diseases have the right to choose the route to parenthood that suits their values and preferences, as long as this does not necessarily create undue burdens or risks for others.^23^ Certainly, conventional egg and embryo donation, like adoption, does not represent a devalued alternative of family building. Rather, the deep interest in procreation is about individual *choice* and interest in being genetically related with one’s child.

There will always be some risks and uncertainties associated with the use of MRT in humans until it is widely implemented in the clinical context and evidence accumulates. Thus, MRT needs to be initially used as a risk reduction treatment for carefully selected patients. Mitochondrial diseases shorten lives, cause severe disability and leave anguish in their wake. MRT has already moved from theory and research into clinical practice, and its responsible use could make a real difference for thousands of families living with mitochondrial disease.

## MRT: Is it a “Therapy”?

Another criticism is based on the notion that the rhetoric of parental autonomy in the context of MRT conceals an economic drive to promote a technology that does not provide a *cure*, because it allows the creation of *new* people, rather than treating *existing* ones: “MRT is not a standard cure or a therapy; rather it helps to create a healthy person who otherwise would not exist. A non-existing child has no interest in being created. We do not have a moral reason to create healthy people for their sake.”^24^

We agree with the criticism that MRT is not a therapeutic intervention for people who have mitochondrial disease. “Therapy” may thus be a misleading description of the technology. MRT is a form of IVF that gives women affected by mitochondrial disease the opportunity to have children free of mitochondrial disease. Still, it is important to bear in mind that there are no proven therapies for mitochondrial disease. With co-factor cocktails and stem cell transplants, one might be fortunate enough to manage symptoms sufficiently well to maintain a reasonable quality of life for a reasonable length of time. However, patients with mitochondrial disease will be hugely impacted by a disease that is, at the very least, life-limiting.^25^

Not everyone considers the alternatives to MRT as viable options.^26^ As such, in the absence of a comprehensive regulatory framework, prospective parents may well seek MRT in jurisdictions with no established regulations. This further exposes them to the associated risks and shortcomings of unregulated practices around the world.^27^ As Norman Daniels argued, the case for a moral right to health care relies on promoting equal opportunity by preventing and curing disease.^28^ Thus, efforts must be made to consider what the clinical translation of this technology would look like from a scientific, ethical, and policy perspective.

Obviously, “a non-existing child has no interest in being created.” The philosophically challenging Non-Identity Problem^29^ aside, society does have a moral duty to those children who are born due to their parents’ choices. If children cannot be harmed by being born with a disease, they may nonetheless be viewed as wronged by being born without a minimal standard of “sufficiency” to which all children are supposedly entitled.^30^ Every child has the right to “the enjoyment of the highest attainable standard of health.”^31^ *Health* is one desirable outcome of socially *just* decision-making that reflects a moral concern with reproductive autonomy, which is central to women and couples’ welfare. *Just* institutions must prioritize the health and overall well-being and interests of children. This would follow the pragmatic reasoning that securing children’s health is a prerequisite to their enjoyment of self-respect, attachment, and autonomy later in life.^32^

## MRT: A “Treatment” for Infertility?

A key area regarding requiring further exploration is the tension surrounding the possible applications of the technology. While the primary aim of MRT is to prevent mitochondrial diseases, the technology could also be used to “treat” infertility. The claim that MRT maybe a new means of solving issues of infertility is based on the notion that oocyte mitochondria are the cause of some cases of infertility.^33^ As such, in Ukraine, Nadiya has advertised MRT as “a unique approach to the treatment of infertility.”.^34^ Also, Greece’s Institute of Life has been conducting a clinical trial led by Greek and Spanish doctors since 2019. This pilot study is “researching multiple IVF failures caused by cytoplasmic dysfunctions of oocytes, and the potential of addressing serious mitochondrial diseases.”^35^

According to critics, encouraging alternative uses of MRT “concerns the common strategy of arguing for the introduction of an ethically controversial technology by insisting on its potential therapeutic benefits and only later defending its potential non-therapeutic uses once it has been successfully introduced.”^36^ Some have argued that the criteria for accessing MRT are grounded mainly in the reproductive autonomy of future parents; thus, “the therapeutic/non-therapeutic moral boundary does not exist” in this context.^37^ However, there is a lack of concrete evidence demonstrating that MRT provides higher live birth rates than standard IVF. The application of MRT as a “treatment” for infertility; thus, remains uncertain.^38^

In a robust regulatory framework, MRT should only be allowed in clearly defined situations of otherwise “serious” mitochondrial disorders. Although important socio-ethical issues revolve around MRT’s clinical translation, the most pertinent of them can be addressed by limiting the use of the technology (i.e., defining a distinct boundary between using MRT to prevent debilitating diseases and “treating” infertility).^39^

## Conclusion

Against the backdrop of the ongoing policy and bioethical debates surrounding MRT, its research and clinical landscapes are rapidly evolving. In identifying the challenges to the clinical implementation of MRT, we should address the legitimacy of using this technology to fulfill the deep interest of many in having and raising genetically related children free of mitochondrial disease. Risk/benefit issues remain one of the prominent arguments against this interest; yet, the importance of addressing the significance of genetic kinship remains underexplored. As a fundamental legal right and bioethical principle, respect for one’s autonomy guides reproductive rights, and should be carefully considered in assessing the risks and benefits of MRT.

MRT could be considered a viable option for women with mitochondrial disease for whom predictive tests (i.e., preimplantation genetic diagnosis and prenatal testing) are likely to be inappropriate, who qualify to use the technology, and want genetically related children free of mitochondrial disease. Still, since MRT is an invasive procedure that requires great skill, it is essential to have the highest level of guidance to ensure all the scientific and clinical conditions are optimized. As stated in the UK’s scientific reports on the safety and efficacy of MRT (2011-2016),^40^ there will always be some risks and uncertainties associated with the use of MRT in humans until it is widely implemented in the clinical context and evidence accumulates. Thus, MRT needs to be initially used as a risk reduction treatment for carefully selected patients.

Mitochondrial diseases shorten lives, cause severe disability and leave anguish in their wake. MRT has already moved from theory and research into clinical practice, and its responsible use could make a real difference for thousands of families living with mitochondrial disease.

## Note

The authors have no conflicts of interest to disclose.
